# To observe or not to observe peers when learning physical examination skills; that is the question

**DOI:** 10.1186/1472-6920-13-55

**Published:** 2013-04-17

**Authors:** Bernard Martineau, Sílvia Mamede, Christina St-Onge, Remy MJP Rikers, Henk G Schmidt

**Affiliations:** 1 Université de Sherbrooke, Faculté de médecine et des sciences de la santé, 3001 12ème avenue nord Sherbrooke, Québec J1H 5N4, Canada; 2 Institute of Psychology, Erasmus University Rotterdam, P.O. Box 17383000 DR, Rotterdam, The Netherlands

**Keywords:** Medical education, Physical examination skills, Skills training, Undergraduate students, Psychomotor skills, Peer observation

## Abstract

**Background:**

Learning physical examination skills is an essential element of medical education. Teaching strategies include practicing the skills either alone or in-group. It is unclear whether students benefit more from training these skills individually or in a group, as the latter allows them to observing their peers. The present study, conducted in a naturalistic setting, investigated the effects of peer observation on mastering psychomotor skills necessary for physical examination.

**Methods:**

The study included 185 2^nd^-year medical students, participating in a regular head-to-toe physical examination learning activity. Students were assigned either to a single-student condition (n = 65), in which participants practiced alone with a patient instructor, or to a multiple-student condition (n = 120), in which participants practiced in triads under patient instructor supervision. The students subsequently carried out a complete examination that was videotaped and subsequently evaluated. Student’s performance was used as a measure of learning.

**Results:**

Students in the multiple-student condition learned more than those who practiced alone (81% vs 76%, p < 0.004). This result possibly derived from a positive effect of observing peers; students who had the possibility to observe a peer (the second and third students in the groups) performed better than students who did not have this possibility (84% vs 76%, p <. 001). There was no advantage of observing more than one peer (83.7% vs 84.1%, p > .05).

**Conclusions:**

The opportunity to observe a peer during practice seemed to improve the acquisition of physical examination skills. By using small groups instead of individual training to teach physical examination skills, health sciences educational programs may provide students with opportunities to improve their performance by learning from their peers through modelling.

## Background

Physical examination (PE) is a core element of physicians’ daily activities and plays an important role in adequate diagnostic performance [1]. Much attention is dedicated, therefore, to the development of medical students’ skills to appropriately perform PE. However, this does not seem to be an easy endeavour because a recent survey showed that a substantial proportion of American clerkship directors consider students to be less prepared than necessary to perform PE when they arrive at the clerkships [2], an opinion shared by the students themselves [3]. To face this challenge, most medical schools have developed specific strategies for teaching PE skills at the undergraduate level [2]. These strategies, built upon theories of motor skills acquisition, mostly involve demonstration of the skill followed by practice with feedback [4]. Great importance is attributed to offering opportunities for students to practice the PE skills extensively. Students often have the chance to practice alone or together with their peers, but it is not known whether students benefit more from individual or group practice. Observation has been recognized as a key strategy to foster motor skills acquisition [5]; however, it is not clear if observation may also play a role in teaching PE skills. If it does, students might benefit from practicing in groups, with an opportunity to observe their peers. The present study investigates whether peer observation has a beneficial influence on the acquisition of PE skills.

The decision to teach PE in groups is often made on the basis of practical considerations. For example, a lack of trainers or simulated patients may compel the school to organise PE teaching in-group sessions. Still, this decision may also be pedagogically sound if one considers research on observational learning. Bandura’s works on learning through modeling [6] advocate that observation provides a template for practice, generating the opportunity to correct one’s own actions, while practicing the skill, based on knowledge of the outcomes. Theories of acquisition of psychomotor skills [7], built upon this construct, suggest that observing a model leads learners to develop a mental blueprint of the to-be-learned task, which acts as a reference while performing the task. Consequently, they use this blueprint to compare it with their own performance while practicing the to-be-learned task [8], which aids in detecting and correcting mistakes. Moreover, Schunk [9] has suggested that watching a peer might help to reinforce one’s self-efficacy, which may positively influence the performance.

Studies in domains outside medicine have provided some evidence in support of the positive influence of observation on learning psychomotor skills. For example, Shea, Wulf and Whitacre [10] demonstrated that practice in dyads, which allows observing a peer practicing the skill, was superior than learning alone to mastering the use of a stabilometer. They concluded that a combination of practice and observation fosters learning more than practicing alone. Aiming at determining if the benefits of learning in dyads derived from observation or from dialogue between participants, Granados and Wulf [11] investigated mastery of cup stacking skills. They demonstrated that participants from conditions in which it is possible to observe another learner learned more, i.e., produced faster movement times and made fewer errors, than participants from conditions that allowed dialogue between peers.

In medicine, the first studies on modelling focused on surgical skills. Custers, Regehr, McCulloch, Peniston, and Reznick [12] confirmed the importance of modelling in showing that observing videos of experts executing a simple surgical procedure (i.e., the excision of a skin lesion and closure of the resulting wound) increased students’ performance compared to those who only read a description of the procedure before executing it. This study also demonstrated that there is no significant advantage of observing more than one model. However, this study investigated the influence of modeling on specific surgical skills, which differ greatly from PE skills. Surgical tasks involve fine, serial, discrete, close motor skills aimed at surgical treatment, taught to more advanced students [13], whereas PE implies serial gross motor skills aimed at searching physical signs for diagnostic clues, usually taught to novices.

The study reported here, conducted in a naturalistic environment, aimed at determining if peer observation while learning physical examination influences the acquisition of psychomotor skills necessary to master an integrated PE skills. We investigated, first, whether observing a peer while practicing PE skills fosters learning in comparison to practicing without observation; and second, whether the number of peers observed (i.e., one or two) during the practice affects acquisition of the to-be-learned skill. It is expected that students observing peers will perform significantly better than students who do not observe peers, but, consistent with previous findings [12], there will be no added value of observing more than one peer.

## Methods

### Setting

This study was conducted at the University of Sherbrooke. The undergraduate medical education program wherein we conducted the experiment is a four-year Problem-Based Learning (PBL) curriculum including an 18-month clinical clerkship. To prepare students for the clerkship, clinical skills are taught through a series of activities within organ-based system modules. More specifically, PE skills are taught at three different moments within the curriculum: PBL sessions; transdisciplinary activities and various PE practice sessions [14]. In PBL sessions, specialist tutors teach in separate, focused sessions, the PE exam of specific organs related to their discipline. They used educational strategies such as demonstration, practice, coaching, and feedback from tutors and peers. During transdisciplinary activities, clinical skills from different disciplines are combined with teaching of communication, clinical reasoning and PE sequence combination learned during PBL sessions. During practice sessions, which take place twice per semester, students practice their PE skills on two to four standardized or real patients, receiving feedback from mentors.

### Ethics approval

Ethics approval was obtained from the Ethic research committee for Education and Social sciences of Sherbrooke University. All participants provided written, informed consent.

### Participants

One hundred and eighty-seven (187) second-year students participated to the study (mean age = 22.1; 73 males). Two students were removed from the analyses due to missing data. We randomly assigned students to one of two learning settings: (1) the single-student condition and (2) the multiple-students condition. In the first setting students practiced alone without the opportunity to observe a peer. In the second setting, students practiced in groups of three, having therefore the opportunity to observe two peers.

### Materials and procedures

#### The learning activity

The task to be learned by the students was a complete head-to-toe PE (excluding neurological and locomotor exam) taught during an activity offered to facilitate the integration of the different components of PE that students had learned separately during their organ-based system modules. At the end of the activity, students are expected to have mastered the sequence of the integrated PE, and to complete it in ten minutes.

This activity, coached by a Patient-Instructor (PI), is regularly offered in the middle of the last semester of the second year of the undergraduate program. PIs are volunteers who work as simulated patients in several educational activities in the undergraduate medical program. Before the present study, the PIs received a 30-hours training on the specific sequences of PE to be coached. The training, provided by the first author, who is a clinician, aimed at standardizing coaching to be provided by the team of patient instructors.

#### Procedure

PIs met participants to teach them the head-to-toe physical examination. Each participant had 45 minutes with a PI, which translated into a 45-minute session for the participants in the single-student condition and a 135-minute session for the participants in the multiple-students condition.

The sequence adopted during the activity was as follows: (1) the PI presented the PE sequence to students; (2) a participant practiced the PE sequence on the PI for 30 minutes, receiving immediate feedback from the PI and from their peers when present; (3) the same participant was videotaped performing the complete PE sequence within 10 minutes; and (4) the participant received final feedback. Steps 2 to 4 were repeated for the other two participants in the multiple-student groups.

#### Measure of performance

Two of the authors (BM, CSTO), through an iterative process, developed a checklist to assess PE participants’ performance (see Additional file 1 for PE components included in the evaluation checklist). They constructed a grid that reflects the skills taught in the curriculum design. The checklist included 158 items divided into four subscales: (1) positioning, assessing whether the student positions his/her body adequately, relative to patient’s position, (2) ordered execution, assessing whether the student respects examination order at each step of PE, (3) gesture precision, assessing whether the student uses the right examination technique and touches the appropriate structure adequately, and (4) procedural efficiency, measuring if the student maximizes the utilization of each patient’s position, thereby minimizing repositioning the patient from one posture (standing-sitting-lying) to another.

Inter-rater reliability (IRR) measured by intra-class correlations (ICC), namely the two-way random model for unique measure, showed reliable results when five raters assessed five different participants’ performance. The ICCs was 0.95. The principal investigator identified areas for improvement in standardization and provided feedback to the PIs on how to adjust their scoring. As the procedure was shown to be reliable, two of the five PIs who participated in the reliability test, scored videos of participant’s performance that we distributed randomly to them. They were blind to the learning activity condition of participants.

### Analysis

We calculated a participant’s performance score by adding all the checklist items and converting this sum into a percentage. To assess the influence of practicing alone with the PI versus practicing in a group, we compared the mean performance score obtained in the single-student condition and the multiple-student condition using an independent t-test.

Based on the two different learning settings, we subsequently assessed the observation condition and a no-observation condition, by grouping participants from the single-student condition with the first participants in the multiple-students condition (i.e., no-observation condition), and grouping the second and third participants from the multiple-students groups (i.e., observation condition). We also used a mean comparison (t-test) to study the influence of observing a peer once versus twice, on performance of PE skills.

To assess the effect of learning activity situation (individual versus group learning), and of number of observations (zero, one or two observations), an ANOVA was used to compare the performance according to the student order (i.e., single-student, first in multiple-student condition, second in multiple-student condition, and third in Multiple-student condition). To estimate the effect sizes, we calculated partial eta-squared for the ANOVA, and correlations for the t-test comparison [15,16]. We used Cohen’s [17] tables to interpret effect sizes.

## Results

### Influence of peer observations on the acquisition of head-to-toe PE skills

Descriptive statistics of students’ performance, for single-student vs. multiple-students conditions, are presented in Table 1. It can be observed that participants in the multiple-students condition had higher scores than participants in the single-student condition group (t[183] = 2.88, p < 0.05, r = 0.21).

**Table 1:** Mean performance scores standard deviations and p-value

	**N**	**Mean**	**SD**	**P-value**
Single-condition	65	76.23	10.64	0.004
Multiple-students condition	120	80.85	10.30	
No observation group*	108	75.90	10.52	< .001
Observation group**	77	83.90	8.92	

* Students from the single condition and the first students of the multiple-students condition formed the “no observation group”.

** The second and third students of the multiple-students conditions formed the “observation group”.

The descriptive statistics for the no-observation versus the observation conditions are presented in Table 1. Participants who observed at least one peer before their own training performed better than students who did not observe peers (t[183] = 5.42, p < 0.05), with a moderate effect size (r = 0.37), suggesting that the higher scores obtained by the multiple-students condition resulted from the opportunity to observe their peers.

### Influence of the number of observations on the acquisition of head-to-toe PE skills

The influence of observing peers when learning PE skills is illustrated in Table 1. Participants’ performances vary according to the order in which they executed the PE. Significant differences were found according to the order of student’s practice, F(3, 181) = 9.78, p < .001, n^2^_p_ = 0.14. Post hoc analyses revealed that differences were significant between the single-student condition and both the second (p = .002) and third (p = .001) student in the multiple-students condition, as well as between the first student in the multiple-students condition and the second (p = .001) and third (p = .001) in the multiple-students condition. There was no difference between the single-student condition and first in the multiple-students condition nor between the second and the third students in the multiple-students condition.

## Discussion

Participants in the single-student condition and the first participants in the multiple-students condition, i.e., those students who did not have the chance to observe any peer practicing the skills, obtained similar scores but did not perform as well as students who observed their peers (i.e., the 2nd and 3rd participants in the multiple-students condition). These results are consistent with our initial hypothesis, and seem to provide a demonstration of the value of observation for the acquisition of psychomotor skills required to execute PE.

All participants were equally exposed to the presentation of the skills by the instructor, and had the same amount of time to practice them before performing the whole PE sequence, which suggests that peer observation determined the observed difference in performance. The mechanisms through which peer observation acted were not investigated in this study, but the results are consistent with theories on observational learning that postulate the potential benefits of using modeling to teach skills. Observing their colleagues practicing the PE may have provided the students in the multiple-students condition with opportunities to refine their perception about the to-be-learned skills, and to discern different aspects of the to-be-executed actions [6]. A similar benefit of observation was found in studies on acquisition of surgical skills [10] and other motor skills [5]. The similarity of scores obtained by the 2^nd^ and the 3^rd^ students within the multiple-students condition indicates that there is at best a limited advantage conferred by observing peers during PE training more than once. A finding that is in line with studies in the surgical field [10].

The effect of the learning condition was only moderate. This moderate effect size could be explained by the fact that those students in the present study were not entirely naive to the to-be-learned skills; they had learned different parts of the PE before the learning activity in which the study was conducted. Consequently, they mainly had to integrate various components of PE that have been previously learned into a head-to-toe examination, which may be different from learning entirely new skills. The performance scores of the participants showed to be high with little room for improvement. Therefore, observing one peer might have been sufficient in this context, but it will need future studies to ensure the amplitude of the size effect in earlier stages of their learning.

We used a detailed grid instead of global ratings to assess the students’ performance. This may be seen as a drawback of the study, because detailed checklists have been shown to fail in discriminating between different levels of expertise [18]. On the contrary, global ratings have been used to reliably assess complex competences, for example in surgery [19]. However, the learning activity in the present study was directed for novice students at the same level of their training. Moreover, it aimed at fostering integration of different components of PE psychomotor skills that had been previously addressed in other regular activities, and a detailed assessment of performance was favoured because it would allow for identifying which of the several components of the psychomotor skills had been acquired. A detailed assessment tool was also considered to correspond to the coaching provided by PIs. It also allows a better standardization of the assessment of student’s performance to be provided by the PIs.

Another limitation is that is not possible to exclude the possibility that the observed effect on performance was due to interaction among peers, which occurs naturally when students practice in-group. Peer feedback could have also contributed to increase the performance, as studies have shown to happen, for example, in writing skills learning. [20] Disentangling the influence of peer feedback from that of peer observation during PE learning requires further investigation.

Ste-Marie and colleagues [5] suggested that future research on observational learning should go beyond experimental designs with a limited number of participants, to be included in regular pedagogical activities. The present study was conducted as part of the regular activities of a medical undergraduate program. The use of a naturalistic setting for the study ensured its feasibility and increases the probability that similar activities will be reproducible in other programs. In addition, the high number of participants would help reducing the selection bias that sometimes occurs in experimental settings. However, the naturalistic setting also poses drawbacks such as an asymmetric design. It was not possible to ensure that the total time on task was equal for each participant, because participants in the multiple-students condition had overall more exposure to the PE so that they could observe their peers. It could be argued, therefore, that instead of deriving from peer observation, the higher performance shown by students in the multiple condition groups was in fact produced by the extra learning time they had. Nevertheless, the fact that there were few additional benefits after observing one peer indicates that there would be limited potential effect of the longer exposure time.

To better understand the value of peer observation when acquiring PE skills, future studies should evaluate if the quality of the peers’ performance being observed influences the acquisition of PE skills. In addition to observation of a peer, it would be interesting to examine whether other factors such as observation of standardized video or feedback provided by peers and instructors provides incremental benefit or produces an effect similar to peer observation. Moreover, this study suggested that peer observation increased performance, but students were tested immediately after the learning activity. It is not known whether the positive effects of peer observation would last, and future studies should explore this issue.

## Conclusions

In summary, the results of this study support the importance of peer observation in the acquisition of psychomotor skills needed to execute the PE. The opportunity to observe peers performing PE within an instructional context may help participants integrating the multiple components of PE. By using small groups instead of individual training to teach PE, health sciences educational programs may provide their students with an opportunity to observe peers performing a task, allowing modelling to take place during learning and favouring performance improvement.

## Competing interests

The authors declare that they have no competing interests.

## Authors’ contributions

BM conceived the design of the learning activity and the study, participated in the data analyses, and drafted the article. SM collaborated in the development of the study design and in drafting and reviewing the manuscript. CSO performed the statistical analyses, participated in the development of the study design and helped to draft the manuscript. RR helped in the development of the study design and helped to draft the manuscript. HS helped to draft the manuscript. He is also the supervisor of BM for his PhD. All authors read and approved the final manuscript.

## Pre-publication history

The pre-publication history for this paper can be accessed here:

http://www.biomedcentral.com/1472-6920/13/55/prepub

## Supplementary Material

Additional file 1 PE components comprised in the evaluation checklist. Click here for file
