# Supplementary material for: To observe or not to observe peers when learning physical examination skills; that is the question
Source: BMC Med Educ. 2013 Apr 17;13:55. doi: 10.1186/1472-6920-13-55 (PMC3637796; doi:10.1186/1472-6920-13-55)
Supplement: Additional file 1 — PE components comprised in the evaluation checklist. [file 1472-6920-13-55-S1.docx]

**Additional file**

**Additional file 1 – Appendix**

PE components comprised in the evaluation checklist

1. General inspection

2. Vital signs: Blood pressure, pulse, and respiratory rate

3. Physical examination of ears, nose, and throat

4. Palpation of cervical lymph nodes

5. Palpation of axillary lymph nodes

6. Lung inspection and auscultation

7. Jugular examination and hepatojugular reflux

8. Cardiac apex

9. Anterior thoracic palpation of sternochondral and costochondral junctions

10. Cardiac and carotids auscultation

11. Inspection and auscultation of abdomen and femoral area

12. Palpation of abdomen and inguinal area

13. Search of abdominal visceromegaly

14. Vascular examination of lower limbs
